# Surviving the nonsurvivable combination of a mycotic aneurysm progressing into a concomitant aorto-bronchial- and aorto-esophageal fistula, a case report

**DOI:** 10.1186/s13019-023-02387-y

**Published:** 2023-10-12

**Authors:** Tim Somers, Bastiaan R. Klarenbeek, Ilse J.E. Kouijzer, Ad F.T.M. Verhagen, Michel W.A. Verkroost

**Affiliations:** 1 Department of Cardio-thoracic Surgery, Radboud University Medical Center, Geert Grooteplein Zuid 10 (Route 615), Nijmegen, 6525 GA Netherlands; 2 Department of Surgery, Radboud University Medical Center, Nijmegen, Netherlands; 3 Department of Internal Medicine and Radboud Centre for Infectious diseases, Radboud University Medical Center, Nijmegen, The Netherlands

**Keywords:** Mycotic aneurysm, Aorto-bronchial fistula, Aorto-esophageal fistula, Survival

## Abstract

Aortic mycotic aneurysms are a rare but life-threatening condition and may be associated with aorto-bronchial- and aorto-esophageal fistulas. Although both very rare, they carry a high mortality and require (urgent) surgical intervention. Surviving all three conditions concomitantly is extraordinary. We describe a patient who underwent staged repair of such combined defects.

Case presentation: A 67-year-old woman suffered from chest pain, dyspnea, and hemoptysis. A CT-scan showed two thoracic aneurysms that rapidly progressed in a pending rupture. Open surgery was not deemed opportune, therefore endovascular repair (TEVAR) was performed. Due to a suspected mycotic nature of the aneurysms accompanied by a severely destroyed left lung, she underwent open repair three days after TEVAR. During this operation, an aorto-bronchial- ánd aorto-esophageal fistula were seen and respectively treated by a pneumonectomy and direct closure of the esophageal perforation, in addition to an aortic interposition graft. Although her condition improved steadily, follow-up CT-scan 20 days after open repair showed a major esophageal defect and possibly bronchopleural fistula. After careful consideration, she underwent a reoperation. A bronchopleural fistula was treated by additional bronchial stump resection and the esophageal wall was closed in two layers after necrotic tissue resection. Additionally, the great omentum was inserted into the thorax to cover all surgical sutures. She was treated with intravenous antibiotic and antifungal therapy, and after three months she was discharged home in good condition. Eight months postoperatively, antibiotic regime was successfully reduced and one year in follow-up she is still relapse free.

Conclusions: The combination of a mycotic aortic aneurysm with an aorta-bronchial- and aorto-esophageal fistula is extremely rare, with a very low chance of survival. Both the bronchial- and esophageal wall have to be eroded by the expanding aneurysm, prior to rupture of the aortic wall, resulting in simultaneous presentation of both fistulas. To the best of our knowledge this condition has only been described previously in two other cases, one of which survived. The success in our case may be due to performing vigorous surgery only after initial stabilization, targeted antibiotic- and antifungal therapy and the use of vital tissue to support definitive healing.

## Background

A mycotic or infected aneurysm, first defined by Sir William Osler in 1885 as an abrupt discontinuation of the aortic wall with the formation of a saccular aneurysm, is a rare condition with a prevalence of 0.6-1.0% of all aortic aneurysms [1, 2]. Early diagnosis and therapy are essential as there is a high risk of rupture [3, 4]. The diagnosis is often made using CT-scan or PET-CT, and most often a coincidental finding. Open surgery is still the gold standard for intervention, although in specific cases endovascular or solely antibiotic therapy are to be considered [5–7]. Still, mycotic aneurysms remain life-threatening, not only because of the high risk of rupture, but also because of the high reported mortality rates [3, 4].

An aorto-bronchial fistula is a rare condition where there is a communication between the aorta and the tracheobronchial tree. This life-threatening condition is seen after endovascular or open thoracic aorta surgery, with reported incidences of 1.5–1.9%, but can also be seen in other aortic disorders like mycotic aneurysms [8]. The main symptom is hemoptysis. Bronchoscopy is the preferred diagnostic modality, although CT gold standard, and open surgical intervention the most desired [8, 9]. The highest mortality rates are described in cases with an aorto-bronchial fistula due to an aortic aneurysm [8].

Another condition related to aortic (mycotic) aneurysms is an aorto-esophageal fistula. This condition is mostly attributed to aortic aneurysms, therapeutic interventions, malignancies or foreign body ingestion [10]. As is the case with aorto-bronchial fistulas, it is a rare and frequently fatal disorder [11]. It requires urgent surgical intervention, where both primary closure and radical esophagectomy have been successful [12].

We present an exceptional case, presented to our department with a mycotic aneurysm that was about to rupture, accompanied by both an aorto-bronchial and aorto-esophageal fistula. Three diagnoses that by itself carry a high morbidity and mortality rate, let alone all three together.

### Case description

**Fig. 1:**
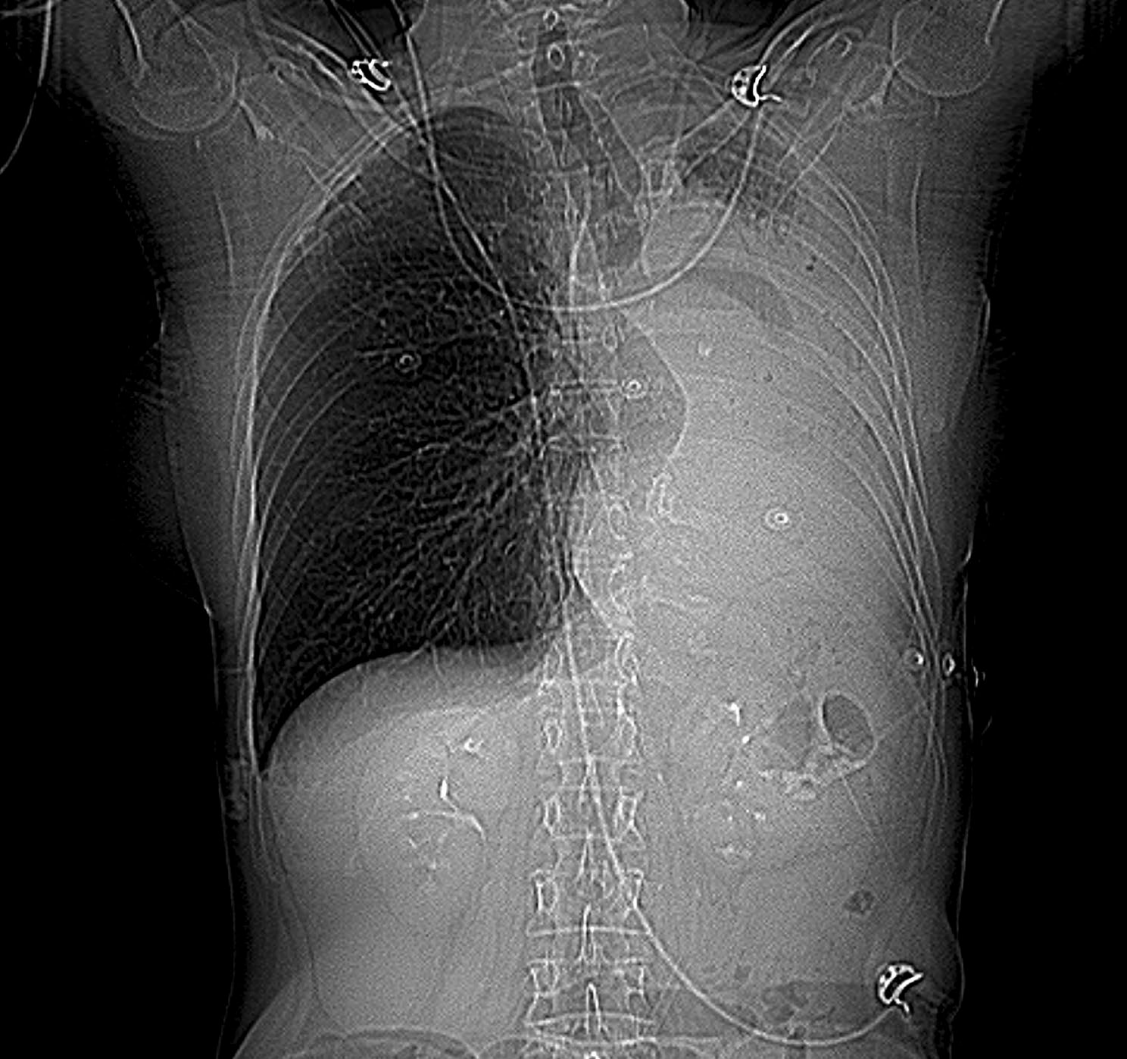
X-ray image prior to CT-scan with no air or pulmonary configuration on the left hemithorax

**Fig. 2:**
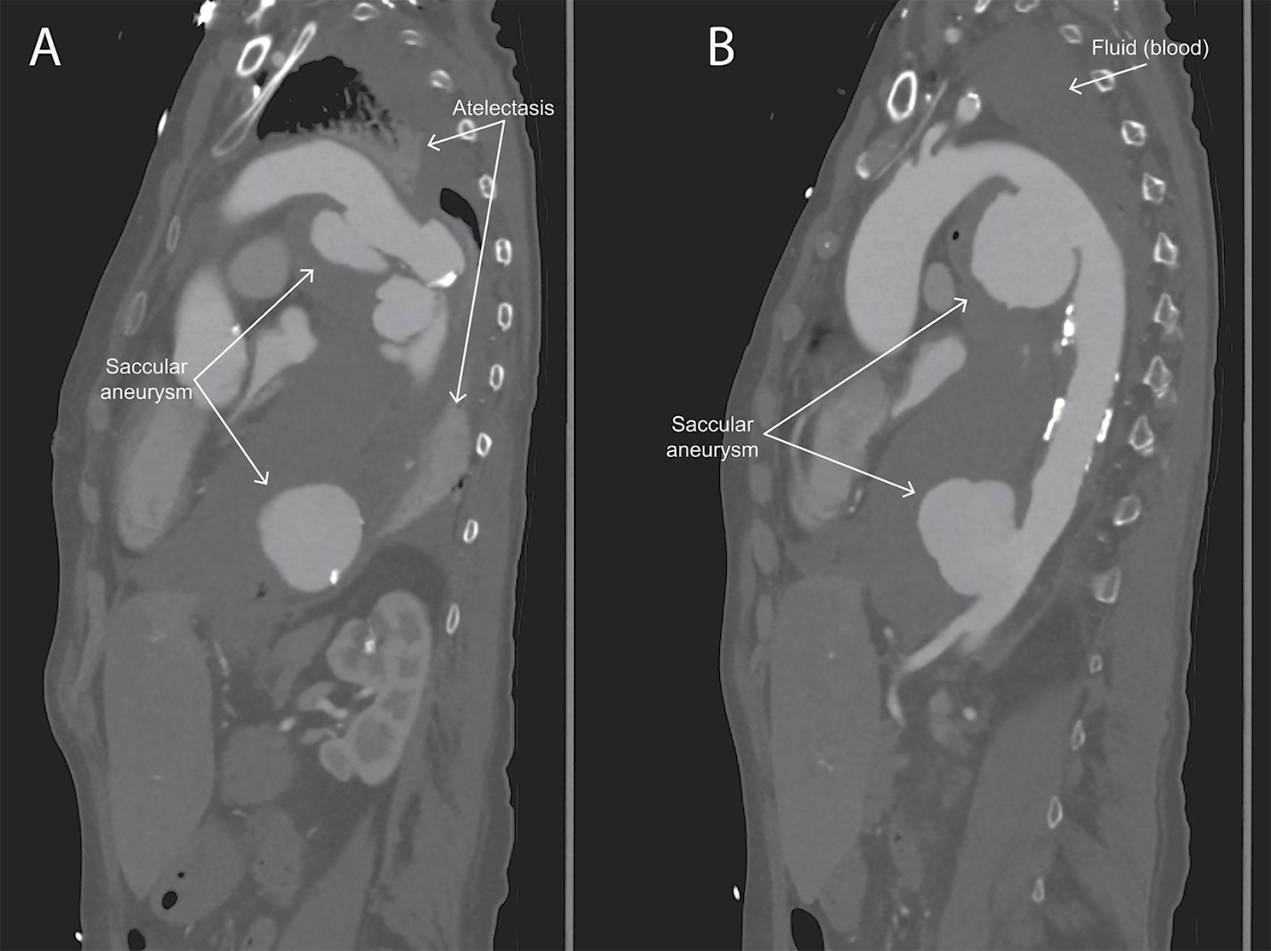
Two sagittal plane CT-scan images of the ascending and descending aorta with the two saccular aneurysms in the proximal (**A**) and distal (**B**) part of the thoracic descending aorta. Also visible is the fluid (blood) that has filled up the whole left hemithorax and replaced the left lung (which is atelectatic)

A 67-year-old woman presented to another clinic with chest pain, dyspnea, and hemoptysis. She had no medical history besides a cellulitis of her lower leg four months earlier and complaints of pyrosis afterwards. A gastrointestinal endoscopy one month prior to presentation showed a mild candida esophagitis for which she was treated with fluconazole. She required oxygen to maintain saturation levels > 95%, had significant reduced breath sounds on the left compared to the right side, and was hypertensive (systolic blood pressure 148mmHg). There were no cardiac murmurs and no fever (temperature 36.8 degrees °C). Laboratory results showed reduced hemoglobin (Hb 6.3 mmol/L), elevated leucocytes (17.3 × 10^9^/L), normal renal function (creatinine 61 µmol/L) and elevated C-reactive protein (CRP 101 mg/L). A CT-scan was performed because of the chest pain and dyspnea and showed two thoracic saccular aneurysms and complete atelectasis of the left lung (Figs. 1 and 2). For surgical intervention she was transferred to our (tertiary) hospital. During this transfer her complaints of thoracic pain increased significantly and therefore a new CT was performed upon arrival. This showed progression of the aneurysm by 7 mm (in only few hours) with active leakage of the most proximal aneurysm towards the esophagus. An open approach, under deep hypothermic arrest due to the anatomical conditions, was thought most suitable as it most probably had an infectious origin based on the CT-scan (air bubbles around aneurysms). However, due to the fast progression and pending rupture with hemodynamic instability, the need for full heparinization in open surgery would probably lead to massive bleeding, before being able to control the aorta, and ultimately death of the patient. Therefore, she underwent an emergency endovascular intervention (TEVAR) as bridge to open surgery. After stabilization with TEVAR, laryngoscopy was performed, but lumen compression prevented the scope from introduction into the left main bronchus.

**Fig. 3:**
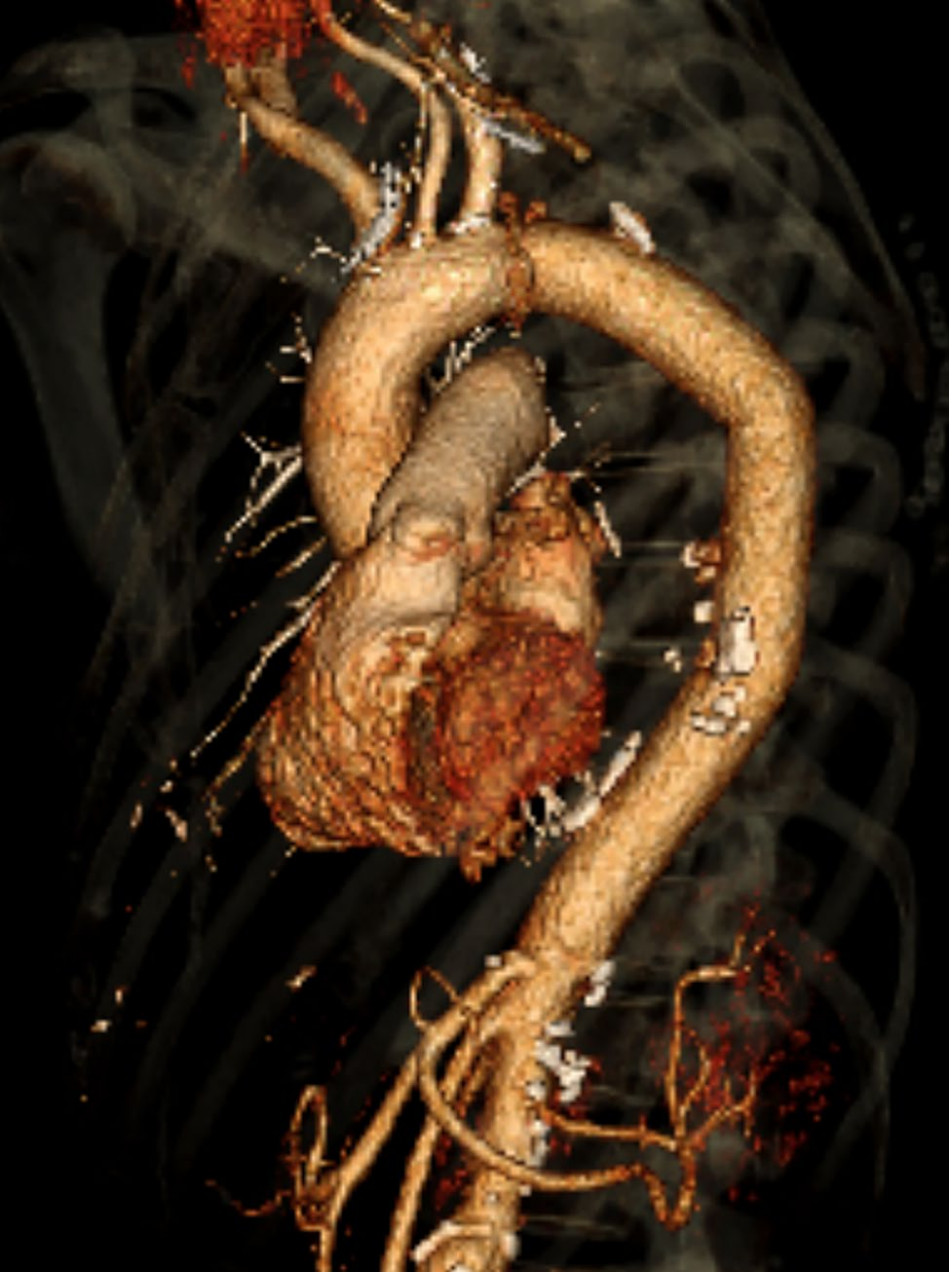
Postoperative 3D reconstruction of the aorta with the interposition graft in place

**Fig. 4:**
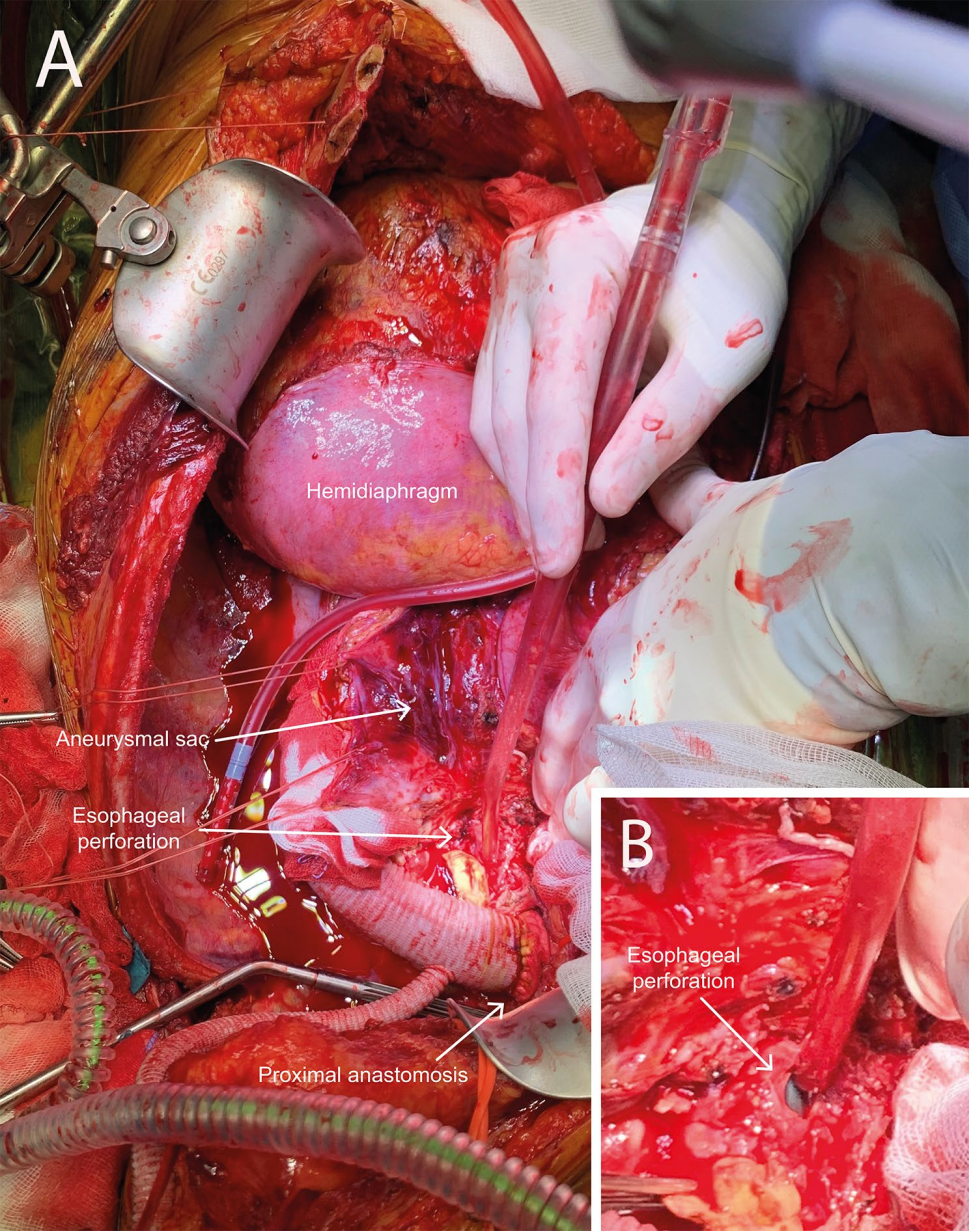
Peroperative situation with the interposition graft in place and the tip of suction device in the esophageal perforation (**A**) and close-up (**B**)

Three days later, in a more stable condition and under broad-spectrum antibiotic therapy, she underwent an open repair in line with most optimal therapy for mycotic aneurysms. Prior to this surgery, blood cultures were taken and returned negative. In these three days, the TEVAR stent graft had the opportunity to adhere preventing leakage after full heparinization in open surgery. However, a longer time interval between TEVAR and open surgery was not deemed opportune for the compressed lung to be saved. In addition, the patient was still critically ill and had multiple aneurysms with potential for rupturing necessitating urgent surgery as decided by a multidisciplinary team. The endoprosthesis was removed and an interposition graft was placed from the left subclavian artery to the coeliac trunk (Fig. 3), under deep hypothermic circulatory arrest for a total of 35 min (22 and 13 min respectively for the proximal and distal anastomosis). Because of erosion of the left main bronchus (an aorto-bronchial fistula) and a partially destroyed lung due to bleeding intraoperatively, a pneumonectomy was performed consecutively. After the pneumonectomy an esophageal defect due to erosion and infectious process came at sight (Fig. 4, an aorto-esophageal fistula), which was primarily closed, followed by the placement of a nasogastric tube. Both erosions were suspected to have been caused by the infection that already had caused the aneurysm formation and were not visible on initial CT-scan, most likely due to inflammatory responses and hematoma formation. Multiple chest tubes were placed in the thoracic cavity and directly clamped. Every 2 h, clamps were released to monitor postoperative bleeding and evacuate any excess air. Within three days postoperative, chest tubes were removed.

Postoperatively, tissue cultures were positive for anaerobic polymicrobial flora and *Candida* spp., most likely secondary to the perforated bronchus and esophagus rather than the primary infectious cause for the aneurysms. *Coxiella burnetii* serology and PCR were negative. The infectious diseases department was consulted to guide the optimal antibiotic therapy. Initially, amoxicillin and ceftriaxone were started empirically, a few days later piperacillin/tazobactam and anidulafungin were started based on the cultures and local protocols.

Her three-week stay at the intensive- and medium care unit was predominantly marked by right sided pleural effusion requiring drainage, delirium, and overall weakness. After these three weeks she was transferred to the wards, however still in a very weak condition.

A CT-scan performed after failure of the swallowing study, showed a major defect (27 mm) in the lateral wall of the esophagus and a defect of the bronchial stump (fistula) confirmed by bronchoscopy. In multidisciplinary consultation with the cardiothoracic surgeon, gastroenterologist, and gastrointestinal surgeon a reoperation was planned to close the bronchopleural fistula and esophageal defect and to cover the mediastinum with the greater omentum, inserted into the thorax by a laparotomy (separately to keep the diaphragm intact). The patient and her family, after thoroughly overthinking the significant risks and possible complications of this reoperation in such a fragile condition, chose to go for this third surgical intervention. In a third stage the bronchial stump was resected and closed by the cardiothoracic surgeon and the esophageal perforation was closed by the gastrointestinal surgeon in two layers followed by great omentum plasty covering both the bronchial stump, esophagus, and vascular prosthesis. Additionally, a jejunostomy was placed.

Cultures collected during the reoperation showed *S. epidermidis* for which vancomycin was added to the current antibiotic regime of piperacillin/tazobactam and anidulafungin. After six weeks of piperacillin/tazobactam this was switched to clindamycin due to increased levels of liver enzymes. Unfortunately, patient appeared allergic to clindamycin (drug eruptions). As clindamycin was seen as the most ideal therapy, including chronic suppressive therapy, a desensitization protocol was performed successfully (amended from: [13]).

Patient recovered well after this reoperation. There were no signs of fever and she regained physical strength after switch from total parenteral to enteral feeding. Three months after the initial admission to our hospital patient was discharged homewards in a remarkable good condition and full enteral intake (normal diet). Antibiotic therapy on discharge consisted of vancomycin, anidulafungin and clindamycin. Three months after the final operation, vancomycin was discontinued, however, because of a successive rise of CRP levels and suspicion of uncontrolled infection, vancomycin was restarted with good clinical response. Six months later, vancomycin was successfully discontinued and chronic suppressive treatment with clindamycin and posaconazole was continued. Current follow-up duration is 1.5 years without any signs of relapse.

## Discussion

A mycotic aneurysm is a rare disease, making it unique when seen together with other rare, even more life-threatening diseases at the same time. Here we present a case of a contained rupture of a mycotic aneurysm that has led to two accompanying diseases with high mortality rates: an aorto-bronchial fistula and an esophageal perforation. Remarkably, this patient survived her condition, was discharged home in an impressively good condition and free from reoperation for over 1.5 years.

Although mycotic aneurysms are rare, early diagnosis is essential due to the high risk of rupture [14]. However mycotic aneurysms are often a coincidental finding on CT, which is the preferred imaging modality [14]. This case is illustrative for the unexpected finding, but also shows that even in a few hours the aneurysm can progress quickly to a pending rupture. PET-CT scan was not performed in this case, due to the acute setting and it was not considered to have any addition to the already indicated operation. The therapy of choice is open surgery combined with antibiotic therapy. In stable cases with small aneurysms antibiotic therapy alone with frequent imaging would be sufficient to perform surgery in the most optimal window [5–7]. However, in acute cases with hemodynamic instability or in inoperable patients, endovascular options should form either a bridge to open surgery or be the final therapy [5–7, 14]. Endovascular interventions have received lots of skepticism because a prosthesis is placed into infected tissue with the risk of graft infection [4, 6]. The guidelines provide similar evidence for endovascular intervention as a bridge to open surgery or definite therapy only in the inoperable patients [6, 12, 15]. This case shows that endovascular stenting should be considered in the acute setting to buy time and stabilize the patient as a bridge to open surgery.

Aorto-bronchial and aorto-esophageal fistulas are associated with significant morbidity and mortality and require operative repair [16, 17]. Let alone having both fistulas at the same time. Only two cases have so far been described where both fistulas and an aortic aneurysm appear concomitantly [16]. The first case did not survive. A probable explanation for the difference in survival could be the more vigorous approach in our patient. The earlier described patient by Eren et al. only underwent aortic surgery and closure of both fistulas using sutures [16]. The infiltrated left upper lobe was left in place. Instead, in our case a pneumonectomy was performed because of the severity of infiltration, reducing the risk of infection and sepsis. The second case did survive surgical intervention of the ruptured aneurysm and aorto-bronchial and aorto-esophageal fistula [18]. Difference here was again less vigorous surgery with only closure of the fistulas with sutures without resection of infected tissue. A more significant difference with this case was the absence of pulmonary infiltration and no new esophageal and pulmonary fistulas in the postoperative phase. Interestingly there was no clear description whether specific tissues were used (e.g. omentum) to prevent formation of new fistulas, although this is common practice when closing bronchial stumps [16, 18]. Due to the magnitude of the first open surgery, it was decided to use viable tissue to cover the fistula repairs in this case, according to current guidelines, but the omental flap technique was deemed futile until the second open surgery.

Aorto-bronchial fistulas are rare, but primarily seen in patients suffering from aortic disorders. Most described is post aortic surgery, either endovascular or open, but are also seen in patients with aortic aneurysms [8, 16]., including mycotic (infected) aneurysms [4]. Previously a case was described in which a patient had an aorto-bronchial fistula with a ruptured thoracic aortic infectious aneurysm [19]. A similar progression in hemoptysis was observed in our patient. In cases with infected aneurysms and aorto-bronchial fistula the hemoptysis is characterized by two phases: at first minimal with hematoma formation and some days later massive hemoptysis after breakthrough of this hematoma.

Apart from aorto-bronchial fistulas, aorto-esophageal fistulas may also be associated with aortic (mycotic) aneurysms [10, 20–22]. A case has been described of a patient developing an aorto-bronchial fistula from a mycotic pseudoaneurysm after the aorto-esophageal fistula was surgically treated months before [20]. This aorto-esophageal fistula was caused by a thoracic aorta aneurysm.

Bronchopleural fistulas are most often seen after pneumonectomy [23]. However, in our patient, the (second) broncho-pleural fistula (together with the empyema of the left pleural cavity) might have been caused by persistent leakage from the esophageal perforation.

## Conclusion

Irrespective of all comorbidities a patient has and the poor prognosis or mortality numbers, there are exceptional cases that survive the nonsurvivable. A patient that is not only diagnosed with a mycotic aneurysm with pending rupture, but also with an aorto-bronchial and aorto-esophageal fistula is able to survive and undergo two major surgeries. In this particular case, the successful outcome may be created by means of a vigorous resection of all infected and necrotic tissue, supported by targeted antibiotic and antifungal therapy plus application of viable tissue, common practice nowadays. Endovascular options may be considered as a bridge to open surgery in case of hemodynamic instability or inoperable patients. If there is suspicion of infection or signs of imminent infections (like in bronchopleural fistula or esophageal perforation) omental flap technique is a good way to improve the overall outcome. This technique could be of important use in broncho- and trachea-esophageal fistulas, however it does not eliminate the risk of future complications in cases with infected (endo)vascular grafts in situ. Shared decision making in a multidisciplinary setting could be important when treating such challenging cases.

## Data Availability

Not applicable.
